# High CTSL2 expression predicts poor prognosis in patients with lung adenocarcinoma

**DOI:** 10.18632/aging.203540

**Published:** 2021-09-23

**Authors:** Junnian Song, Jindong Jiang, Na Wei, Zongsheng Duan, Gang Wang, Weiyun Pan

**Affiliations:** 1 Department of First Operating Room, The First Hospital of Jilin University, Changchun 130021, China; 2 Department of Thoracic Surgery, The First Hospital of Jilin University, Changchun 130021, China; 3 Department of Anesthesiology, The First Hospital of Jilin University, Changchun 130021, China; 4 Department of Second Operating Room, The First Hospital of Jilin University, Changchun 130021, China; 5 Department of Intensive Care Unit, The First Hospital of Jilin University, Changchun 130021, China

**Keywords:** CTSL2, lung adenocarcinoma, prognostic biomarker, nomogram, gene set enrichment analysis

## Abstract

Cathepsin like 2 (CTSL2) is a lysosomal cysteine protease, and may be associated with tumor metastasis. However, CTSL2 has not been reported as a biomarker in lung adenocarcinoma (LUAD). In this study, bioinformatics analysis using data from The Cancer Genome Atlas was performed. Wilcoxon rank-sum test and chi-square test were carried out. Kaplan-Meier and Cox regression were performed to evaluate the effect of CTSL2 expression in the overall survival. Our results indicated that CTSL2 in tumor was significantly higher than that in normal tissue (*P* < 0.001). High CTSL2 expression was significantly associated with age (*P* = 0.02), vital status (P < 0.001), and T classification (*P* = 0.03), and correlated with poor overall survival (HR = 1.62, 95% CI = 1.21–2.18, *P* = 0.001). CTSL2 expression was an independent risk factor for overall survival in patients with LUAD (HR = 1.52, 95% CI = 1.12–2.05, *P* = 0.006). A nomogram was plotted for illustration of CTSL2 expression on the risk of LUAD. Furthermore, *in vitro* cell experiments showed the CTSL2 promoted the proliferation and migration of A549 cells. In summary, high CTSL2 expression predicts poor prognosis in patients with LUAD.

## INTRODUCTION

Lung cancer is malignant with high incidence and mortality all over the world [1, 2]. The 5-year survival rate of lung cancer is 4–17% based on different stages when diagnosed [3]. Lung adenocarcinoma (LUAD) is the most common type of lung cancer in histology [1, 2].

The pathogenesis of LUAD is complicated [4]. In addition to genetic factors, the main risk factors are smoking, asbestos, radon and other environmental factors [5]. Although the targeting and immunotherapy of LUAD have made great progresses in recent years, its prognosis is still poor [6]. The main reason is the polymer heterogeneity of lung cancer [7]. Since the current diagnostic methods relying on low-dose CT scans and classic serum tumor markers are limited and not specific, LUAD is usually at an advanced stage when diagnosed [8, 9]. Therefore, it is greatly significant to carry out more in-depth explorations of LUAD, and to find new biomarkers related to its diagnosis and prognosis.

Metastatic progression is one of the biggest challenges, which limits the effect of cancer therapies. Proteolysis is involved in the invasion by cleavage of proteins that mediate adherence to neighboring cells. Cathepsins are a family of lysosomal proteases involved in proliferation, invasion and metastasis of different kinds of cancers [10]. The human cysteine cathepsin family is comprised of eleven members including Cat B, C, F, H, L, K, O, S, V, W, and X/Z, which shares a conserved active site. CTSL2 gene encodes cathepsin like 2 (cathepsin L2, also known as cathepsin V) [11]. CTSL2 is a lysosomal cysteine protease, and may be associated with tumor metastasis [10, 12]. Overexpression of CTSL2 has been found in various human cancers, including breast cancer, squamous cell carcinoma, thymic carcinoma, et al. [13–15]. Besides, CTSL2 is regarded as a potential drug target [16]. These findings indicate that CTSL2 may be a biomarker for cancer diagnosis and prognosis. Therefore, we focus on the role of CTSL2 in LUAD.

In this study, bioinformatics analysis of CTSL2 in LUAD was performed. The association between CTSL2 expression and clinical features in LUAD was studied. CTSL2 expression in the overall survival and on the risk of LUAD was illustrated. Furthermore, *in vitro* cell experiments of CTSL2 expression on cell proliferation and migration of LUAD cells were carried out. CTSL2 is found to have predictive value, and high CTSL2 expression is in association with poor prognosis of LUAD.

## RESULTS

### Characteristics of patients with lung cancer

Totally, 517 LUAD patients were involved, including 277 (53.58%) females, and 240 (46.42%) males (Table 1). As for LUAD stage, there were 277 (53.58%) in I, 122 (23.60%) in II, 84 (16.25%) in III, and 26 (5.03%) in IV. Moreover, the T2 (53.77%), N0 (64.41%), and M0 (67.12%) showed the highest percentage. It can be seen that 99.61% were primary lung cancer.

**Table 1:** Clinical characteristics of the lung adenocarcinoma patients.

**Characteristics**	***N* (%)**
Age (years)	
<55	71 (13.73)
> = 55	427 (82.59)
NA	19 (3.68)
Gender	
Female	277 (53.58)
Male	240 (46.42)
Stage	
I	277 (53.58)
II	122 (23.60)
III	84 (16.25)
IV	26 (5.03)
NA	8 (1.55)
T classification	
T1	170 (32.88)
T2	278 (53.77)
T3	47 (9.09)
T4	19 (3.68)
TX	3 (0.58)
N classification	
N0	333 (64.41)
N1	96 (18.57)
N2	74 (14.31)
N3	2 (0.39)
NX	11 (2.13)
NA	1 (0.19)
M classification	
M0	347 (67.12)
M1	25 (4.84)
MX	141 (27.27)
NA	4 (0.77)
Radiation therapy	
No	400 (77.37)
Yes	60 (11.61)
NA	57 (11.03)
Residual tumor	
R0	345 (66.73)
R1	13 (2.51)
R2	4 (0.77)
RX	25 (4.84)
NA	130 (25.15)
Vital status	
Deceased	187 (36.17)
Living	330 (63.83)
Sample type	
Primary tumor	515 (99.61)
Recurrent tumor	2 (0.39)

Abbreviations: NA: not available; X represents uncertain.

### High CTSL2 expression in LUAD

**Figure 1:**
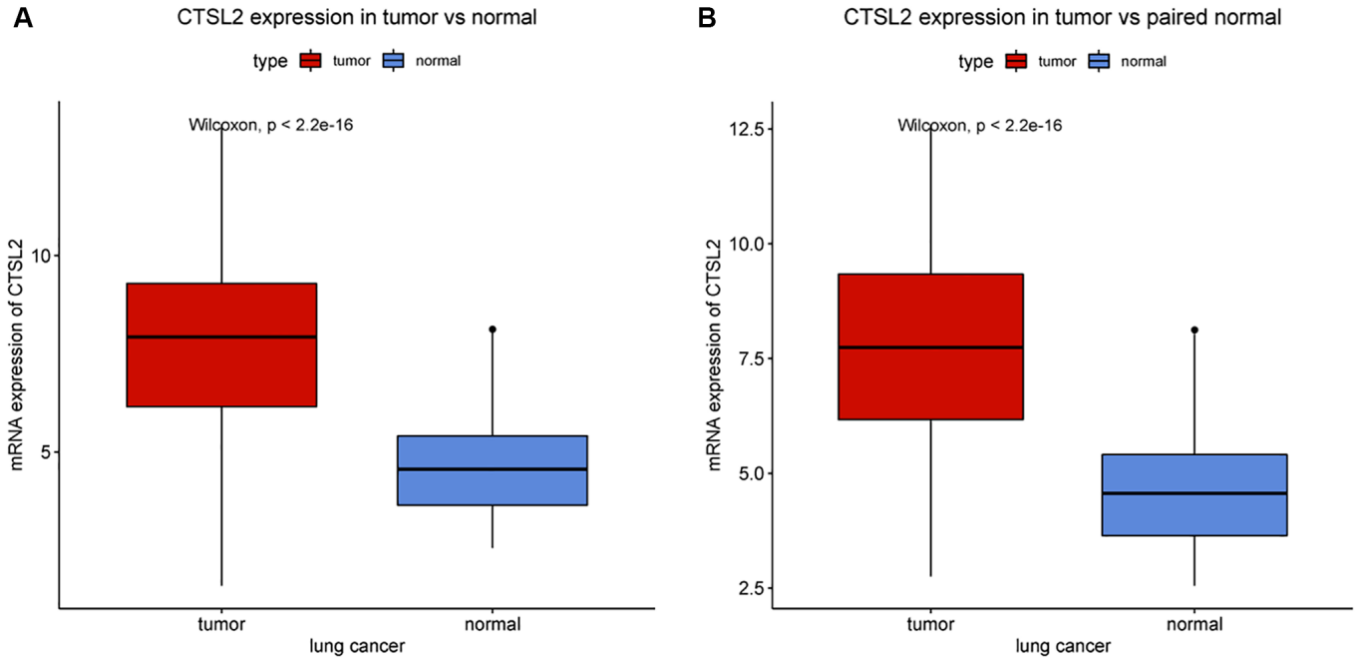
**CTSL2 expression in lung adenocarcinoma tissues.** (**A**) CTSL2 expression in normal and tumor tissues. (**B**) CTSL2 expression in paired tissues.

The expression of CTSL2 in lung adenocarcinoma and normal lung tissues were compared (Figure 1A). The tumor CTSL2 level was significantly increased (*P* < 0.001) in comparison with normal tissue. The comparison of tumor and paired normal tissue further verified the high CTSL2 expression in LUAD (*P* < 0.001; Figure 1B).

### Correlation between CTSL2 expression and clinical characteristics

**Figure 2:**
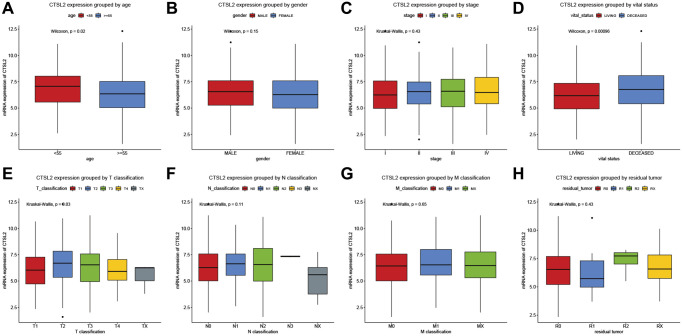
CTSL2 expression of patients with lung adenocarcinoma grouped by (**A**) age, (**B**) gender, (**C**) stage, (**D**) vital status, (**E**) T classification, (**F**) N classification, (**G**) M classification, and (**H**) residual tumor.

As shown in Table 2 and Figure 2, high CTSL2 expression was significantly associated with age (*P* = 0.02), vital status (*P* < 0.001), and T classification (*P* = 0.03). Meanwhile, gender, stage, N and M classification, and residual tumor showed no significant differences.

**Table 2:** Logistic analysis of the association between CTSL2 expression and clinical characteristics.

**Characteristics**		**Total**	**High**	**Low**	**χ^2^**	**P**
Age	<55	71	42 (17.65)	29 (11.15)	3.771	0.052
	> = 55	427	196 (82.35)	231 (88.85)		
Gender	Female	277	123 (50.2)	154 (56.62)	1.882	0.170
	Male	240	122 (49.8)	118 (43.38)		
Stage	I	277	123 (50.62)	154 (57.89)	2.778	0.423
	II	122	64 (26.34)	58 (21.8)		
	III	84	43 (17.7)	41 (15.41)		
	IV	26	13 (5.35)	13 (4.89)		
T classification	T1	170	67 (27.35)	103 (37.87)	11.503	**0.019**
	T2	278	147 (60)	131 (48.16)		
	T3	47	24 (9.8)	23 (8.46)		
	T4	19	7 (2.86)	12 (4.41)		
	TX	3	0 (0)	3 (1.1)		
N classification	N0	333	149 (60.82)	184 (67.9)	10.893	**0.023**
	N1	96	55 (22.45)	41 (15.13)		
	N2	74	37 (15.1)	37 (13.65)		
	N3	2	2 (0.82)	0 (0)		
	NX	11	2 (0.82)	9 (3.32)		
M classification	M0	347	164 (67.21)	183 (68.03)	0.210	0.902
	M1	25	13 (5.33)	12 (4.46)		
	MX	141	67 (27.46)	74 (27.51)		
Radiation therapy	No	400	179 (84.83)	221 (88.76)	1.222	0.269
	Yes	60	32 (15.17)	28 (11.24)		
Residual tumor	R0	345	173 (89.18)	172 (89.12)	1.733	0.677
	R1	13	5 (2.58)	8 (4.15)		
	R2	4	3 (1.55)	1 (0.52)		
	RX	25	13 (6.7)	12 (6.22)		
Vital status	Deceased	187	107 (43.67)	80 (29.41)	10.746	**0.001**
	Living	330	138 (56.33)	192 (70.59)		
Sample type	Primary tumor	515	243 (99.18)	272 (100)	0.614	0.433
	Recurrent tumor	2	2 (0.82)	0 (0)		

X represents uncertain.

### High CTSL2 expression is an independent risk factor for overall survival

**Figure 3:**
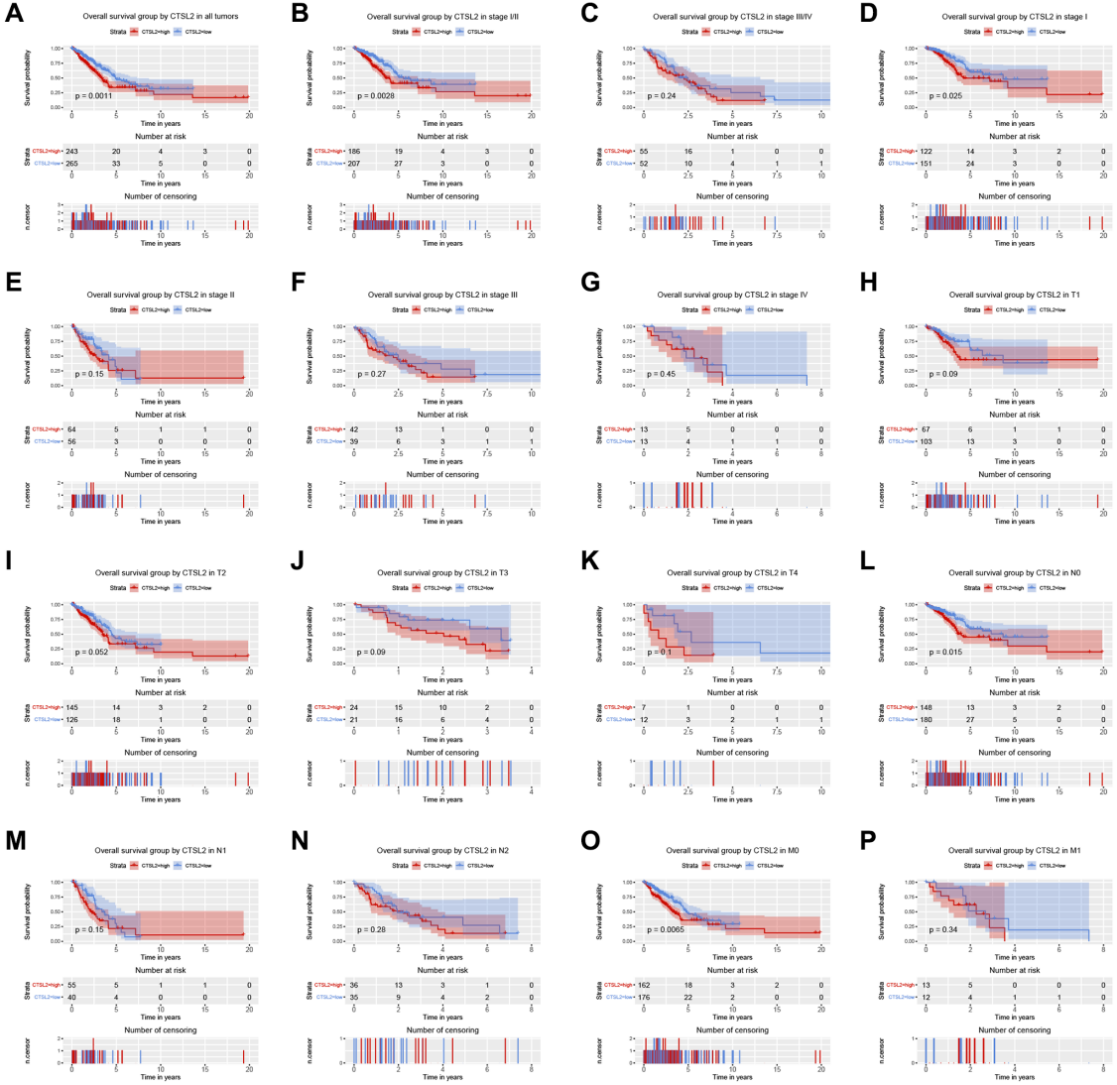
**Kaplan-Meier curve for overall survival in lung adenocarcinoma.** (**A**) CTSL2 in all tumors; (**B**–**G**) Subgroup analysis for stage I/II, III/IV, I, II, III, and IV; (**H**–**P**) Subgroup analysis for T1, T2, T3, T4, N0, N1, N2, M0, and M1.

**Figure 4:**
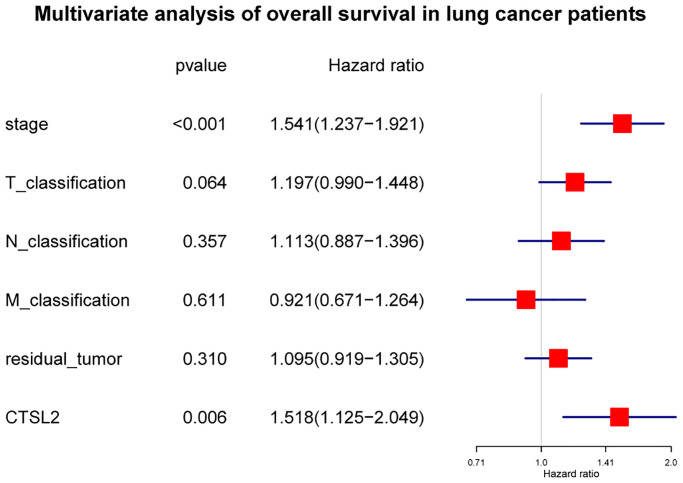
Forest plot of the multivariate Cox regression analysis in lung adenocarcinoma.

As shown in Figure 3A, high expression of CTSL2 was associated with poor prognosis (*P* = 0.0011). The subgroup analysis (Figure 3B–3P) showed that high expression of CTSL2 was significantly associated with poor prognosis in stage I (*P* = 0.025), N0 (*P* = 0.015), and M0 (*P* = 0.0065). As shown in Table 3, the high expression of CTSL2 was significantly correlated with poor overall survival (HR = 1.62, 95% CI = 1.21–2.18, *P* = 0.001). As shown in Table 3 and Figure 4, high CTSL2 expression was confirmed to be an independent risk factor for overall survival (HR = 1.52, 95% CI = 1.12–2.05, *P* = 0.006).

**Table 3:** Univariate and multivariate Cox regression analyses of clinical characteristics associated with overall survival.

**Characteristics**	**Univariate analysis**			**Multivariate analysis**		
	**HR**	**95% CI**	***P***	**HR**	**95% CI**	***P***
Age	0.82	0.55–1.22	0.322			
Gender	1.05	0.78–1.40	0.760			
Stage	1.68	1.47–1.93	**<0.001**	1.54	1.24–1.92	**<0.001**
T classification	1.52	1.26–1.82	**<0.001**	1.20	0.99–1.45	0.064
N classification	1.67	1.41–1.97	**<0.001**	1.11	0.89–1.40	0.357
M classification	1.40	1.04–1.90	**0.029**	0.92	0.67–1.26	0.611
Radiation therapy	1.25	0.89–1.75	0.199			
Residual tumor	1.20	1.01–1.42	**0.037**	1.10	0.92–1.30	0.310
CTSL2 expression	1.62	1.21–2.18	**0.001**	1.52	1.12–2.05	**0.006**

Abbreviations: HR: hazard ratio; CI: confidence interval.

### Diagnostic value of CTSL2 for LUAD

**Figure 5:**
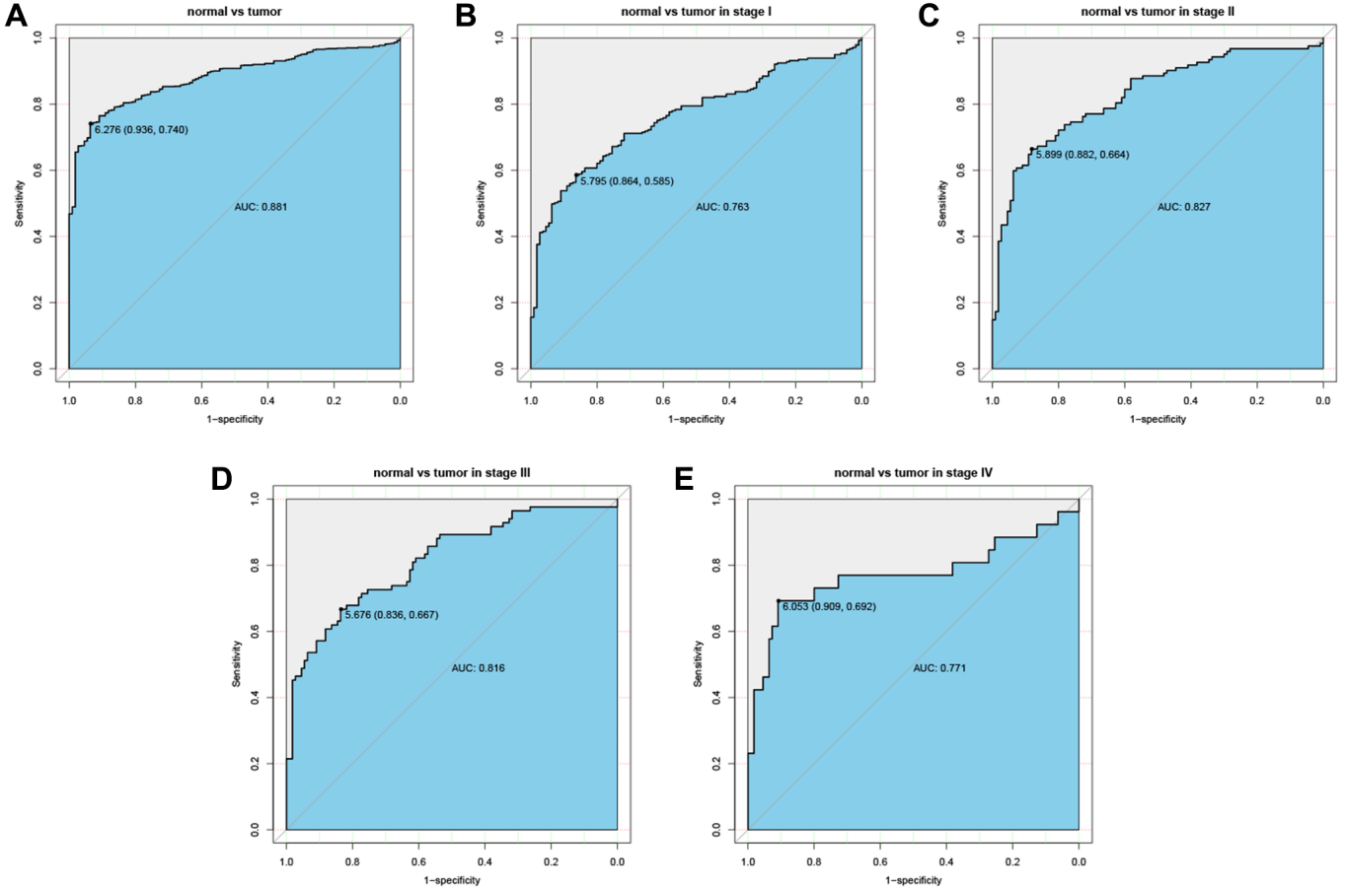
**Diagnostic value of CTSL2 expression in lung adenocarcinoma.** (**A**) ROC curve for CTSL2 in normal lung tissue and tumor; (**B**–**E**) Subgroup analysis for stage I, II, III, and IV.

**Figure 6:**
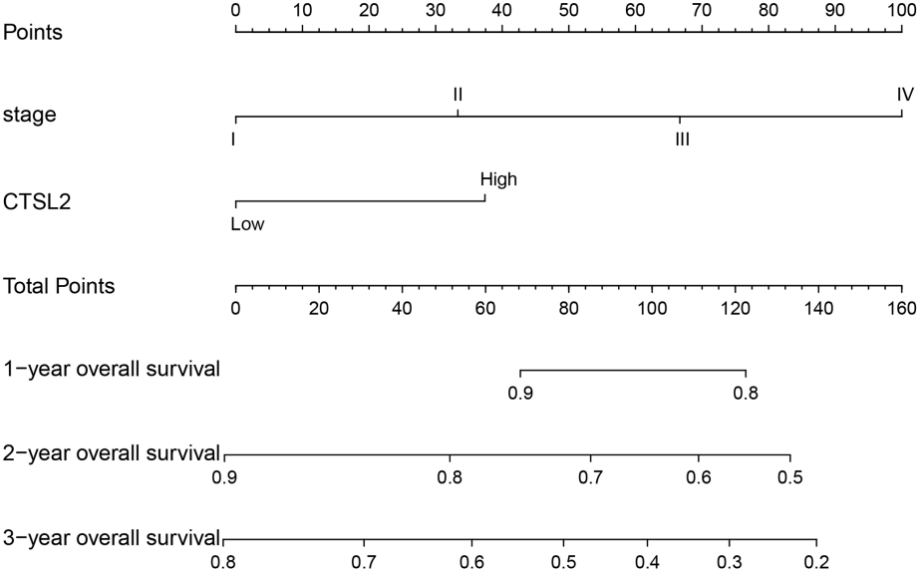
**Nomogram for predicting probability of patients with 1-, 2- and 3-year overall survival.** 1-, 3- and 5-year related survival probabilities were obtained by draw a line straight down to the Risk axis.

The ROC curves were drawn to examine the diagnostic value of for LUAD. The area was 0.881, indicating a modest diagnostic value of CTSL2 expression (Figure 5A). As shown in Figure 5B–5E, the AUC values were 0.763 for stage I, 0.827 for stage II, 0.816 for stage III, and 0.771 for stage IV, respectively. Furthermore, a nomogram model was established for predicting the survival probability of LUAD patients in different years, which involved stage and CTSL2 expression (Figure 6). To evaluate the discrimination and performance of the model, DCA, calibration, and ROC curves were plotted, indicating that the nomogram was stable in predicting the prognosis of LUAD patients (Supplementary Figure 1).

### CTSL2-related signaling pathways by GSEA

**Figure 7:**
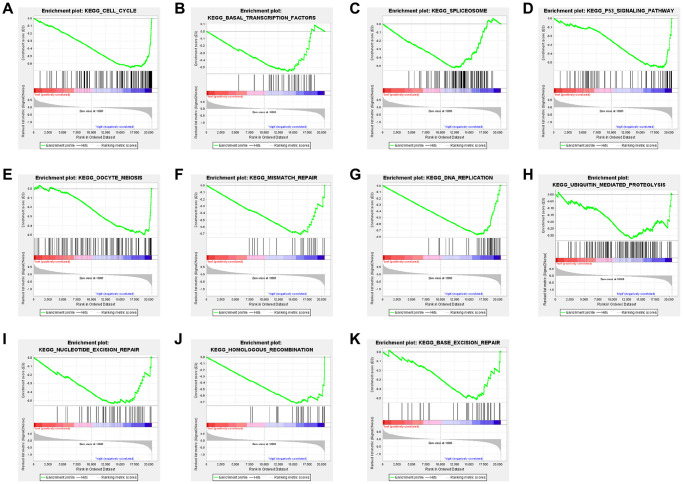
Enrichment plots from GSEA of (**A**) cell cycle, (**B**) basal transcription factors, (**C**) spliceosome, (**D**) p53 signaling pathway, (**E**) oocyte meiosis, (**F**) mismatch repair, (**G**) DNA replication, (**H**) ubiquitin mediated proteolysis, (**I**) nucleotide excision repair, (**J**) homologous recombination, and (**K**) base excision repair in lung adenocarcinoma cases with high CTSL2 expression.

The gene set enrichment analysis (GSEA) was carried out to identify differentially activated signaling pathways in low and high expression of CTSL2. As shown in Table 4, the significant gene sets enriched in the high CTSL2 expression were observed. As shown in Figure 7A–7K, different enrichments were observed in cell cycle, basal transcription factors, spliceosome, p53 signaling pathway, oocyte meiosis, mismatch repair, DNA replication, ubiquitin mediated proteolysis, nucleotide excision repair, homologous recombination, and base excision repair.

**Table 4:** Gene sets enriched in the high CTSL2 expression phenotype.

**Gene set name**	**Size**	**ES**	**NES**	**NOM *p*-value**	**FDR *q*-value**
KEGG_CELL_CYCLE	118	–0.654	–1.935	<0.001	0.103
KEGG_BASAL_TRANSCRIPTION_FACTORS	35	–0.557	–1.909	<0.001	0.069
KEGG_SPLICEOSOME	114	–0.522	–1.894	0.008	0.055
KEGG_P53_SIGNALING_PATHWAY	67	–0.561	–1.829	0.002	0.079
KEGG_OOCYTE_MEIOSIS	112	–0.499	–1.795	0.006	0.081
KEGG_MISMATCH_REPAIR	23	–0.714	–1.781	0.002	0.078
KEGG_DNA_REPLICATION	36	–0.769	–1.770	0.002	0.073
KEGG_UBIQUITIN_MEDIATED_PROTEOLYSIS	134	–0.323	–1.753	0.019	0.074
KEGG_NUCLEOTIDE_EXCISION_REPAIR	44	–0.533	–1.724	0.022	0.085
KEGG_HOMOLOGOUS_RECOMBINATION	26	–0.723	–1.627	0.006	0.146
KEGG_BASE_EXCISION_REPAIR	33	–0.515	–1.580	0.046	0.161

Abbreviations: ES: enrichment score; NES: normalized enrichment score; NOM: nominal; FDR: false discovery rate.

### Validation using independent external database

**Figure 8:**
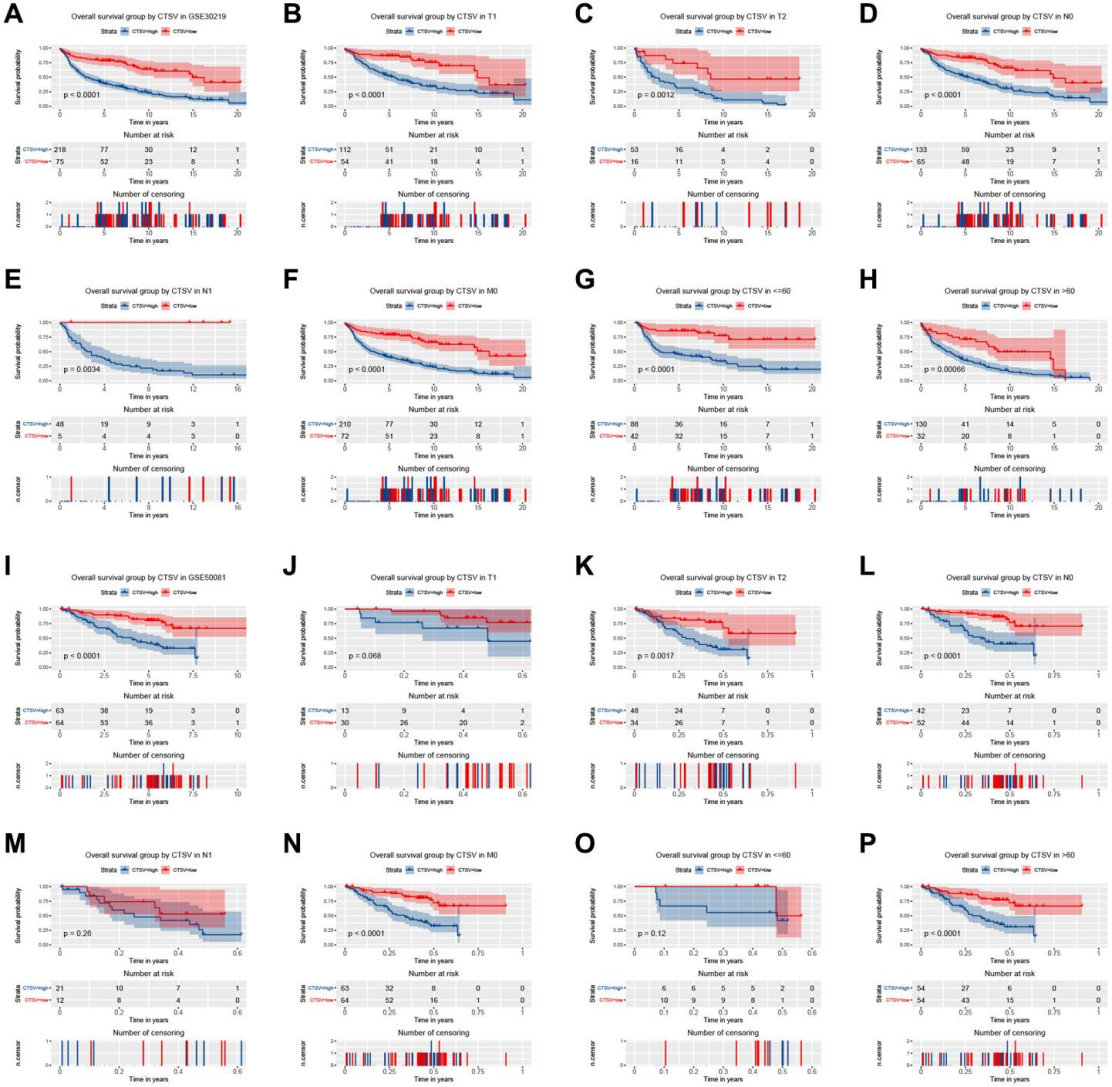
Kaplan–Meier curve for overall survival in lung adenocarcinoma in the validation datasets GSE30219 (**A**–**H**) and GSE50081 (**I**–**P**).

**Figure 9:**
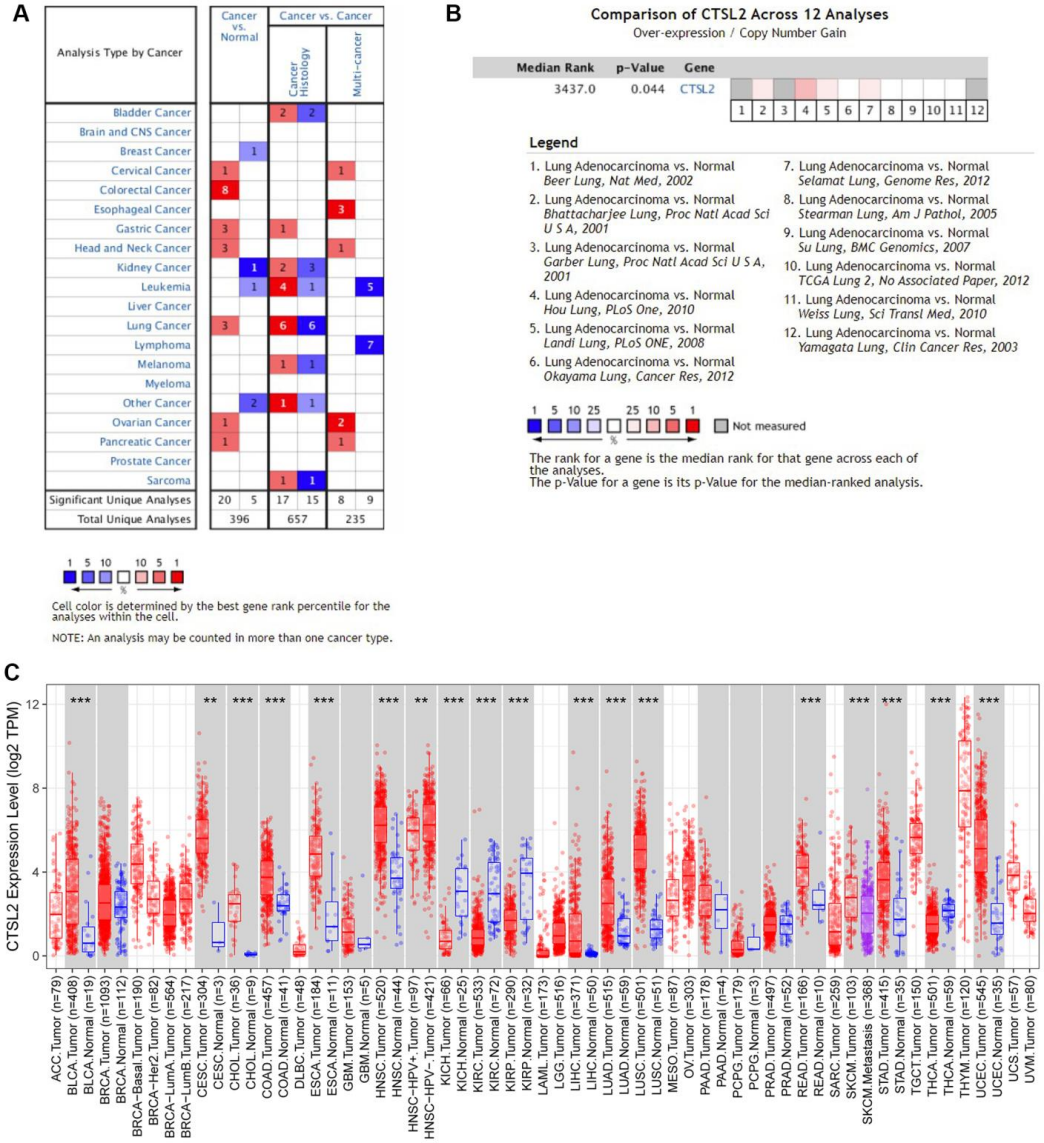
**Expression analysis of CTSL2 by Oncomine and TIMER databases.** (**A**) Expression of CTSL2 in different types of human cancers in the Oncomine database; (**B**) CTSL2 is over-expression (red) in lung adenocarcinoma by Oncomine meta-analysis comparing with normal tissue; (**C**) Expression of CTSL2 in different types of human cancers in the TIMER database.

Two independent external datasets were used to validate the prognostic value of CTSL2 expression. By analyzing GSE30219, we found patients with high CTSL2 expression showed decreased overall survival (Figure 8A–8H). Consistently, decreased overall survival was also observed in patients with high CTSL2 expression by analyzing GSE50081 (Figure 8I–8P). Furthermore, 12 distinct LUAD datasets (including Beer Lung, Bhattacharjee Lung, Garber Lung, Hou Lung, Landi Lung, Okayama Lung, Selamat Lung, Stearman Lung, Su Lung, TCGA Lung, Weiss Lung, and Yamagata Lung) indicated the CTSL2 expression was significantly increased by pooled analysis in the Oncomine database (Figure 9A–9B) [17–27]. Besides, significantly increased CTSL2 expression was observed in the TIMER database (Figure 9C). Of note, high expression of CTSL2 is usually found in different kinds of solid tumors, including lung cancer, cervical carcinoma, cholangiocarcinoma, colorectal cancer, et al.

### High CTSL2 expression in LUAD tissue and cell

**Figure 10:**
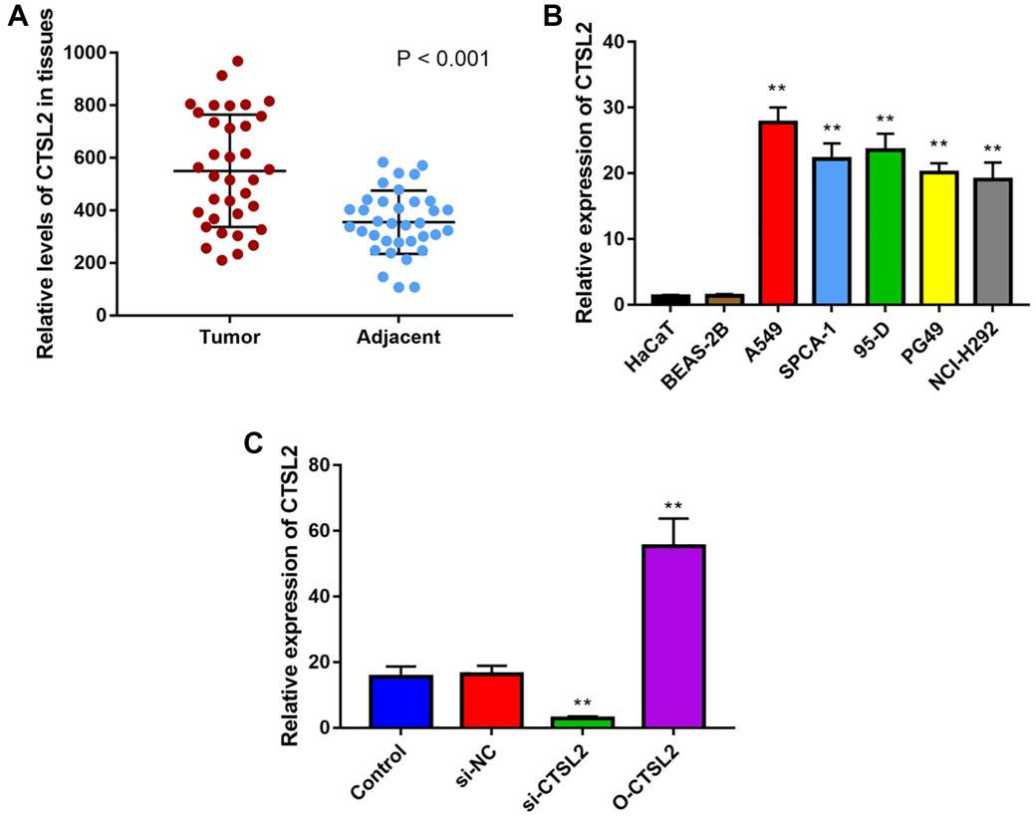
**CTSL2 expression in human LUAD tissues and cell lines.** (**A**) Expression of CTSL2 in 35 LUAD tissues and adjacent normal tissues by qRT-PCR; (**B**) CTSL2 expression levels in HaCaT, BEAS-2B, A549, SPCA-1, 95-D, PG-49, and NCI-H292 by qRT-PCR; (**C**) CTSL2 expression in A549 cells transfected with control, si-NC, si-CTSL2, and O-CTSL2 by qRT-PCR. ^**^P < 0.01.

As shown in Figure 10A, CTSL2 expression was significantly higher in LUAD than adjacent normal tissue (*P* < 0.001). Besides, the CTSL2 expression was significantly increased (*P* < 0.01) in lung cancer cell lines in comparison with normal cell lines (Figure 10B). Notably, A549 cell line showed the highest CTSL2 expression among all the lung cancer cell lines, and used for the subsequent experiments.

### CTSL2 promoted the proliferation and migration of A549 cells

The function of CTSL2 on A549 cell proliferation was studied by transfection of siRNA and non-silencing RNA sequences. As shown in Figure 10C, the qRT-PCR was performed to validate the over-expression and knockdown efficiency. It can be observed that CTSL2 expression was significantly decreased after transfection of si-CTSL2 (*P* < 0.01), and increased after transfection of O-CTSL2 in A549 cells (*P* < 0.01).

**Figure 11:**
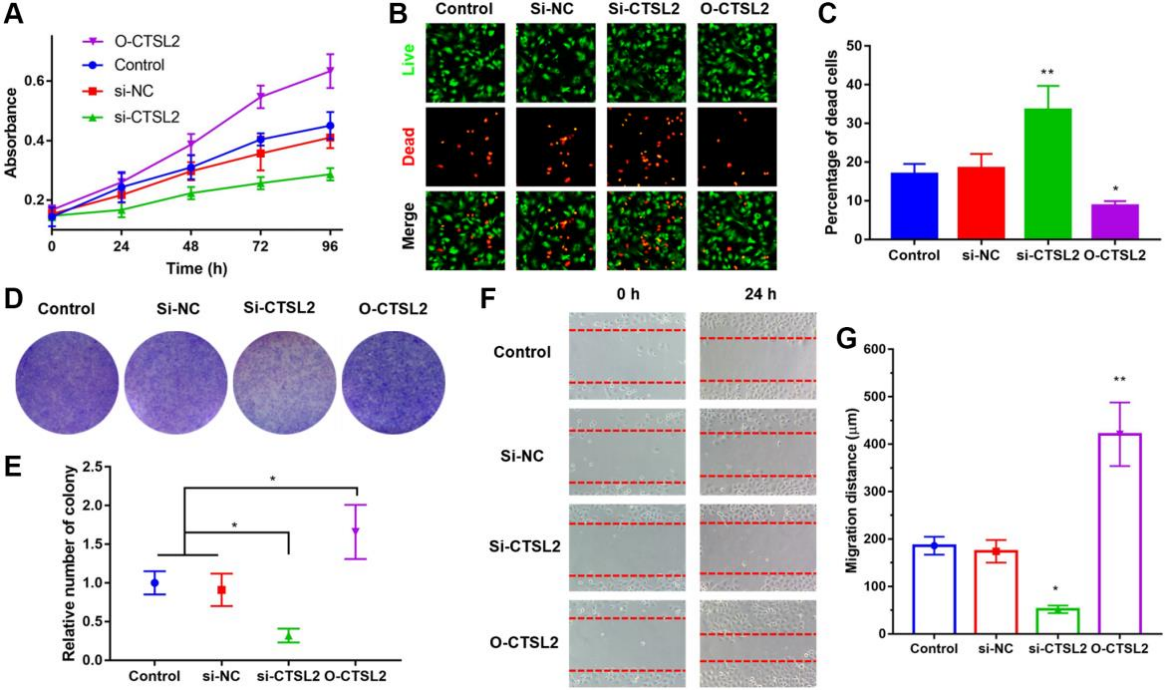
**CTSL2 promoted cell proliferation and migration of LUAD cells.** (**A**) CCK-8 proliferation curve of A549 cells; (**B**) Co-staining of calcein AM and PI of A549 cells, the live cells were stained with green fluorescence, and the dead cells were stained with red fluorescence; (**C**) Percentage of dead cells in different groups; (**D**) Colony formation of A549 cells; (**E**) Relative number of colonies in different groups; (**F**) Cell migration of A549 cells during 24 h; (**G**) Migration distance in different groups. ^*^P < 0.05, ^**^P < 0.01. All the experiments were repeated for three times.

The role of CTSL2 on proliferation and migration of A549 cells was studied. As shown in Figure 11A, over-expression and knockdown of CTSL2 promoted and inhibited the proliferation of A549 cells, respectively. The results of CCK-8 were verified by the LIVE/DEAD staining (Figure 11B–11C). Compared with control and si-NC group, si-CTSL2 group showed significantly higher percentage of dead cells (*P* < 0.01). The results of colony formation were consistent with those of CCK-8 and LIVE/DEAD staining (Figure 11D–11E). Over-expression of CTSL2 showed significantly increased number of colonies (*P* < 0.05). The cell migration assay showed that si-CTSL2 significantly reduced and O-CTSL2 significantly increased the migration distance of A549 cells (Figure 11F–11G).

## DISCUSSION

Exploration of biomarker have been performed in cancer such as breast cancer and liver cancer [28–30]. However, not too much progress has been made in LUAD. We first demonstrated that CTSL2 was highly expressed in LUAD, which was significantly associated with age, vital status, and T classification. Moreover, CTSL2 showed the moderate diagnostic value for LUAD. Data mining has been emerged as an approach to find novel biomarkers [31, 32]. The existed biomarkers have not shown satisfied diagnostic and prognostic value [33–35]. Therefore, it is necessary to explore novel biomarkers to further solve this problem [36–37]. The thymidine kinase 1 was found to improve its diagnostic value for LUAD when combined with carcinoembryonic antigen [38]. Moreover, newly reported biomarkers including uridine-cytidine kinase 2 and long non-coding RNA XLOC_009167 have shown potential value in prognosis [39–40]. Together with these findings, our finding that high CTSL2 expression predicts poor prognosis in patients with LUAD also contributes to the biomarker exploration for clinical practice.

CTSL2 belongs to the cathepsins family, which are involved in proliferation, invasion, and metastasis of different kinds of cancers. Upregulation of cathepsin L protein was observed in the conditional K-ras^G12D^ mouse LUAC model [41]. High CTSL2 expression was found in squamous cell carcinoma by high-density oligonucleotide microarray [14]. mRNA level of CTSL2 is significantly increased in endometrial cancer especially in G3 tumors, indicating it may lead to the progression of endometrial cancer [42]. In our study, we found high CTSL2 expression in LUAD tissue and cell by performing bioinformatic analysis and q-PCR. Similarly, Michael et al. reported the prognostic significance of CTSL2 in breast ductal carcinoma *in situ* [43]. Moreover, our GSEA analysis suggested that high CTSL2 expression may be associated with cell cycle, basal transcription factors, spliceosome, p53 signaling pathway, oocyte meiosis, et al. The phenomenon that CTSL2 promoted the proliferation and migration of A549 cells may be associated with these biological processes and signaling pathways, which needs to be investigated in the future.

In conclusion, high expression of CTSL2 was found in LUAD and associated with clinical progression. High CTSL2 expression was an independent risk factor for OS in LUAD patients. CTSL2 promoted the proliferation and migration of LUAD cells. CTSL2 may serve as a biomarker for diagnosis and prognosis of LUAD. High CTSL2 expression predicts poor prognosis in patients with LUAD.

## MATERIALS AND METHODS

### Data mining

Public TCGA (The Cancer Genome Atlas) database was analyzed for data mining with no ethical concern, involving 517 LUAD patients. The RNA expression of CTSL2 was box-plotted. ROC (Receiver operating characteristic) curve was drawn with the AUC (area under curves) calculated using pROC package [44]. The high-expressed and low-expressed groups were determined by identified CTSL2 threshold level. The survival package in R and Cox model was used as previously reported [45]. The covariates included in the multivariate Cox regression model were stage, T, N, M classification, residual tumor, and CTSL2 expression. GSE30219 (analyzing 293 lung tumor and 14 normal lung specimen) and GSE50081 (analyzing 181 Stage I and II non-small cell lung carcinoma) were used for external validation. Evaluation of subgroups was performed as well. GSEA was carried out for identification of CTSL2 expression-related genes and examination of the survival significances. Oncomine database and TIMER database were used for validation of CTSL2 expression.

### Sample collection

We collected LUAD and adjacent tissues from 35 patients. The samples were kept in liquid nitrogen immediately after resection, and stored at −80°C. The study was approved by the First Hospital of Jilin University Ethics Committee and conformed to the Declaration of Helsinki.

### Cell culture and cell transfection

BEAS-2B, HaCaT, A549, SPCA-1, 95-D, NCI-H292 and PG-49 cell lines were purchased from American Tissue Culture Collection. BEAS-2B, A549, 95-D, and NCI-H292 and were cultured in Dulbecco’s modified Eagle’s medium supplemented with 10% fetal bovine serum. HaCaT, SPCA-1, and PG-49 were cultured in Roswell Park Memorial Institute-1640 supplemented with 10% fetal bovine serum. Antibiotics penicillin and streptomycin (1 %) were used. The cells were cultured at 37°C with 5% CO_2_. The lentiviral vector containing siRNA targeting CTSL2 was constructed. As previously reported, the transfection of four groups including (a) si-CTSL2: si-CTSL2 transfected group, (b) O-CTSL2: CTSL2 overexpressed group, (c) si-NC: an empty lentiviral vector transfected group as negative control, (d) control: un-transfected group. The validation of over-expression and knockdown efficiency was performed using qRT-PCR.

### qRT-PCR

The total RNA extraction was carried out following the instruction of manufacturer (Invitrogen, Thermo Fisher Scientific, USA). Then, the RNA was reversely transcribed into cDNA using the kit (Roche, Basel, Switzerland). The qRT-PCR was performed and the expression of CTSL2 was quantified using 2^−ΔΔCt^ method. The primers were as follows (5′-3′): CTSL2 forward primer, GAAGTCAGAAAGGAAGTACAGAGG; CTSL2 reverse primer, CTCTCCAGTCAACAGATCGTG; β-actin forward primer, ACCCCAAAGCCAACAGA; β-actin reverse primer, CCAGAGTCCATCACAATACC.

### Cell proliferation assay

First, plasmid was added into each plate, followed by culturing for 24 h. After adding CCK-8 solution (10 μL), the cells were placed for 20 min. The 490 nm absorbance was measured, and the cell viability was calculated. Calcein AM and PI co-staining and colony formation assay were further performed [44].

### Wound healing assay

Calcein AM was used to stain the live cells [44]. The cell migration was recorded using fluorescence microscope, and the migration distance was calculated.

### Statistical analysis

R version 3.5.2 package and ggplot2 package in R were used for bioinformatics analysis [45–46]. The Wilcoxon rank-sum test and Kruskal-Wallis test were used. The chi-squared test and Fisher's exact test were used for assessing association. Kaplan-Meier and Cox regression were performed to evaluate the effect of CTSL2 expression in the overall survival [47]. GSEA was performed using data from TCGA. The student’s *t*-test (unpaired, two-tailed) was used to analyze experiment data. *P* < 0.05 was used as the threshold of statistical significance.

## Supplementary Materials

Supplementary Figure 1
