# Supplementary material for: High CTSL2 expression predicts poor prognosis in patients with lung adenocarcinoma
Source: Aging (Albany NY). 2021 Sep 23;13(18):22315–31. doi: 10.18632/aging.203540 (PMC8507295; doi:10.18632/aging.203540)
Supplement: Supplementary Figure 1 [file aging-13-203540-s001.pdf]

SUPPLEMENTARY FIGURE

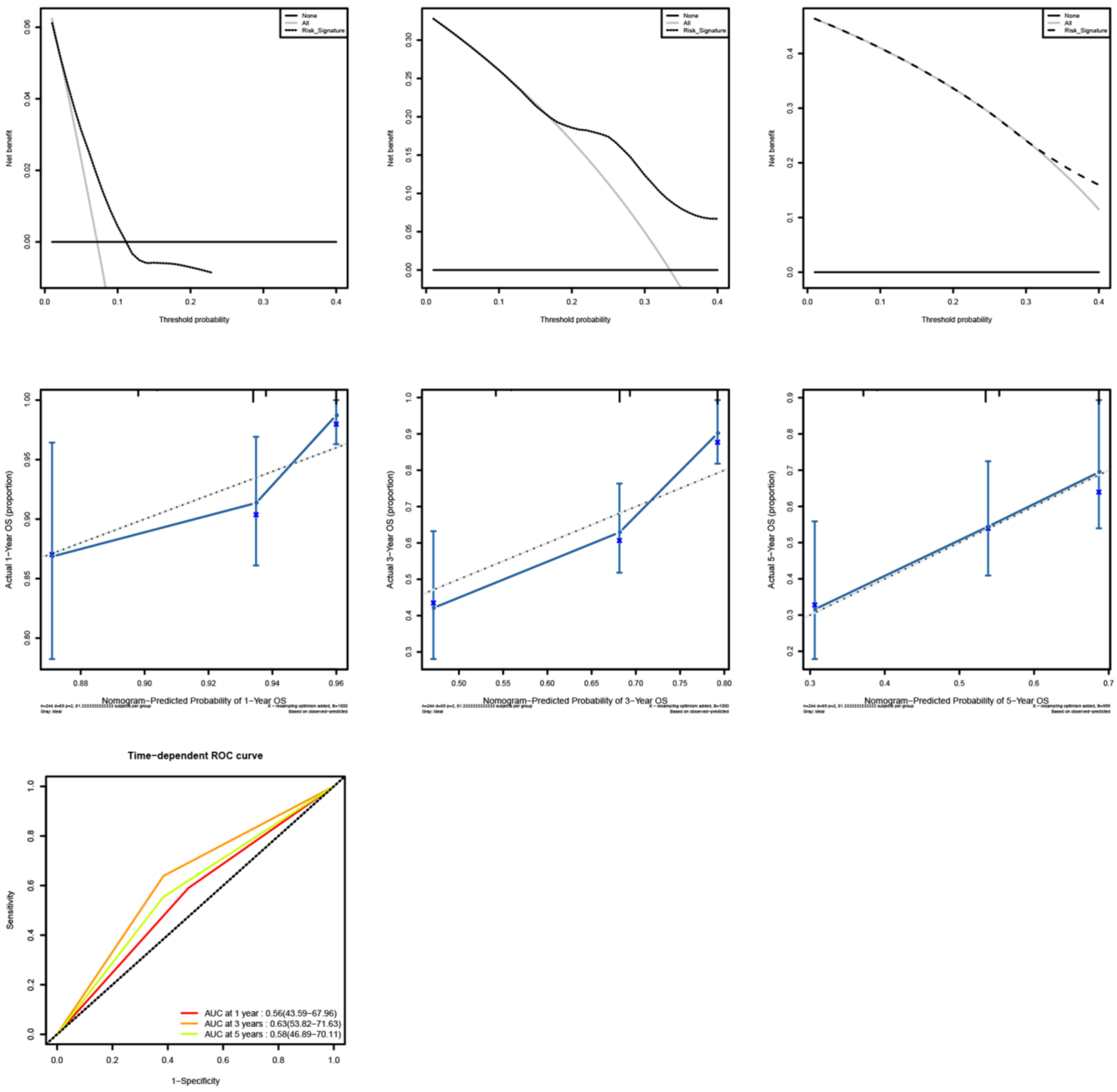

Supplementary Figure 1. Evaluation of the discrimination and performance of the nomogram model. DCA (top panel), calibration plots (middle panel) and ROC (bottom panel) of the nomogram for the probability of OS at 1, 3 and 5 years.
